# Androgen deprivation therapy and radiotherapy in intermediate-risk prostate cancer: A systematic review and meta-analysis

**DOI:** 10.3389/fendo.2022.1074540

**Published:** 2023-01-17

**Authors:** Jiuzhou Chen, Yan Yuan, Miao Fang, Youqi Zhu, Xueqing Sun, Yufei Lou, Yong Xin, Fengjuan Zhou

**Affiliations:** ^1^ Department of Radiation, the Affiliated Hospital of Xuzhou Medical University, Jiangsu, China; ^2^ Department of Cancer Institute, Xuzhou Medical University, Xuzhou, Jiangsu, China; ^3^ Department of Radiation, the Second Affiliated Hospital of Xuzhou Medical University, Xuzhou, Jiangsu, China

**Keywords:** prostate cancer, androgen deprivation therapy, radiotherapy, intermediate-risk, systematic review and meta-analysis

## Abstract

**Objectives:**

Androgen deprivation therapy combined with radiotherapy for intermediate-risk prostate cancer is still a matter of debate. We conducted a meta-analysis to evaluate the necessity of androgen deprivation therapy combined with radiotherapy for intermediate-risk prostate cancer patients.

**Methods:**

A comprehensive literature search of articles was performed in PubMed, Embase, Cochrane library, Web of Science, Chinese National Knowledge Infrastructure, Chinese Biological Medicine, Wanfang, and VIP Databases published between February 1988 and April 2022. Studies comparing the survival of patients diagnosed with intermediate-risk prostate cancer who were treated with androgen deprivation therapy combined with radiotherapy or radiotherapy alone were included. Data were extracted and analyzed with the RevMan software (version 5.3) and the Stata software (version 17).

**Results:**

Six randomized controlled trials and nine retrospective studies, including 6853 patients (2948 in androgen deprivation therapy combined with radiotherapy group and 3905 in radiotherapy alone group) were enrolled. Androgen deprivation therapy combined with radiotherapy did not provide an overall survival (HR 1.12, 95% CI 1.01-1.12, p=0.04) or biochemical recurrence-free survival (HR 1.23, 95% CI 1.09-1.39, P=0.001) advantage to intermediate-risk prostate cancer patients.

**Conclusion:**

Androgen deprivation therapy combined with radiotherapy did not show some advantages in terms of overall survival and biochemical recurrence-free survival and radiotherapy alone may be the effective therapy for intermediate-risk prostate cancer patients.

**Systematic review registration:**

https://inplasy.com/inplasy-2022-8-0095/, identifier 202280095.

## 1 Introduction

Prostate cancer (PC) is the second most common cancer and the fifth leading cause of cancer death among men worldwide. According to relevant epidemiological statistics, there were 1.4 million new cases of PC and 375,000 deaths in 2020 (1). In 2021, PC has the highest 5-year relative survival rate(98%) in the United States, where PC is the most frequent cancer which accounted for 26% of all incident cases in men (2). The 5-year survival rate of early PC can be close to 100% after radical surgery, but PC often has no obvious symptoms in the early stage, and most patients have distant metastasis when diagnosed, and the 5-year survival rate drops to 32% (3). It follows then that today PC still constitutes a global health problem.

At present, the screening and diagnosis of PC mainly rely on prostate-specific antigen (PSA), digital rectal examination, and prostate needle biopsy (4). However, the poor specificity of PSA leads to the over-diagnosis and treatment of PC and the invasive examination brings physical and mental pain to patients as well as increases the economic burden (5). Anti-cancer therapy is rarely based on a single drug and almost always requires combinative approaches since simultaneously attacking more than one target usually achieves greater efficacy (6). It is suspicious whether over-diagnosis contribute to over-treatment. However, radiotherapy (RT) alone may not be effective for treating PC, and RT combined with androgen deprivation therapy (ADT) is often a requisite (7–9). For low-risk PC, RT alone has shown high clinical response but for intermediate and high-risk PC, ADT is necessary for clinical effects.

Current studies are almost based on intermediate and high-risk PC and rarely aim at intermediate-risk PC alone (10, 11). Intermediate risk PC is the most heterogeneous of the three D’Amico risk stratification groups, and because of this, it brings considerable treatment challenges for clinical workers (12). The current National Comprehensive Cancer Network (NCCN) guidelines reclassify intermediate-risk PC patients into favorable intermediate risk (FIR) and unfavorable intermediate risk (UIR) (13). For FIR patients, some trials indicated RT alone is adequate but for UIR patients, ADT is warranted for patients receiving RT and considered for patients receiving RT with brachytherapy boost (14). However, the use of ADT in FIR patients is still controversial (15–17). Considering the potential side effects of over-treatment for patients, we should better define subgroups of patients who may benefit from the combined therapy. Therefore, in this study, we compared the efficacy and safety of RT alone with RT plus ADT in intermediate PC through meta-analysis, to provide evidence-based evidence for the treatment of intermediate-risk PC patients.

## 2 Materials and methods

This article was written in strict accordance with the Preferred Reporting Items for Systematic Reviews and Meta-Analysis (PRISMA) and the protocol for this systematic review was registered on INPLASY (202280095) and is available in full on inplasy.com (https://inplasy.com/inplasy-2022-8-0095/) (18).

### 2.1 Search strategy and study selection

The articles published in PubMed, Embase, Cochrane library, Web of Science, Chinese National Knowledge Infrastructure (CNKI), Chinese Biological Medicine (CBM), Wanfang, and VIP Databases between February 1988 and April 2022 were systematically searched and meanwhile, the related trials in the International Clinical Trial Registry Platform (ICTRP) and the Chinese Clinical Registry up to April 2022 and some conference summaries were also searched and selected. The search terms included (‘Prostatic Neoplasms’ or ‘Prostate Cancer’), (‘intermediate risk’), (‘androgen deprivation therapy’ or ‘endocrine therapy’), and (‘Radiotherapy’ or ‘radiation’).

### 2.2 Inclusion and exclusion criteria 

The inclusion criteria were as follows: clinical trials and retrospective studies directly comparing RT alone with ADT plus RT, all patients were confirmed as PC by histopathological or cytological examination and met the diagnostic criteria of intermediate-risk PC, and sufficient data could be extracted for analysis.

The exclusion criteria were as follows: review articles, systematic evaluations, animal basic experiments or case reports; repetitive articles, studies including no relevant or incomplete data; some ongoing clinical trials with no published results; and violation of any of the above inclusion criteria.

### 2.3 Quality assessment and risk of bias

Randomized controlled trials (RCTs) and retrospective studies were all enrolled in our systematic review and meta-analysis which we assessed the quality of through the Cochrane Collaboration's tool and the Newcastle–Ottawa scale (NOS) respectively. The scoring standard of the Cochrane Collaboration's tool contained the random sequence generation, allocation concealment, blinding of outcome assessment, incomplete outcome data, selective reporting, and other biases. The NOS mainly included the selection (0–4 stars), comparability (0–2 stars), and outcome (0–3 stars). If studies’ scores ≥ 6 stars, it would be regarded as high quality and enrolled in our meta-analysis.

### 2.4 Data extraction and collection

Data extraction and paper collection were independently and carefully performed by two authors (JC and YY), including the first author’s name, year of publication, number of patients, age of patients, interventions, ADT regimen, radiotherapy dose, and so on. If they had differences, a third author (MF) would resolve the discrepancy and determined whether the article would be included. The primary outcome was OS. The secondary endpoints were BCRFS. The hazard ratio (HR) for OS and BCRFS of patients undergoing combined was used for meta-analysis. We used two methods for data extraction of HR. For one thing, we directly extracted HR and its 95% CI from the enrolled articles. For another, by utilizing the methods of Tierney et al, we calculated HR as well as its 95% CI through the figures from the text and data given in the articles.

### 2.5 Statistical analysis

We used the random-effects model through the inverse variance method to calculate the pooled HR and evaluated the heterogeneity of all studies through the Cochrane’s Q test and I^2^ statistic, of which, significant heterogeneity was P<0.1, and I^2^>50.0%. We evaluated the potential publication bias by funnel plots, Egger’s test, and Begg’s test. All P values were two-sided, and P<0.05 was considered to manifest statistical significance. All statistical analyses were performed using the RevMan software (version 5.3) and the Stata software (version 17).

## 3 Results

### 3.1 Characteristics of studies

**Figure 1:**
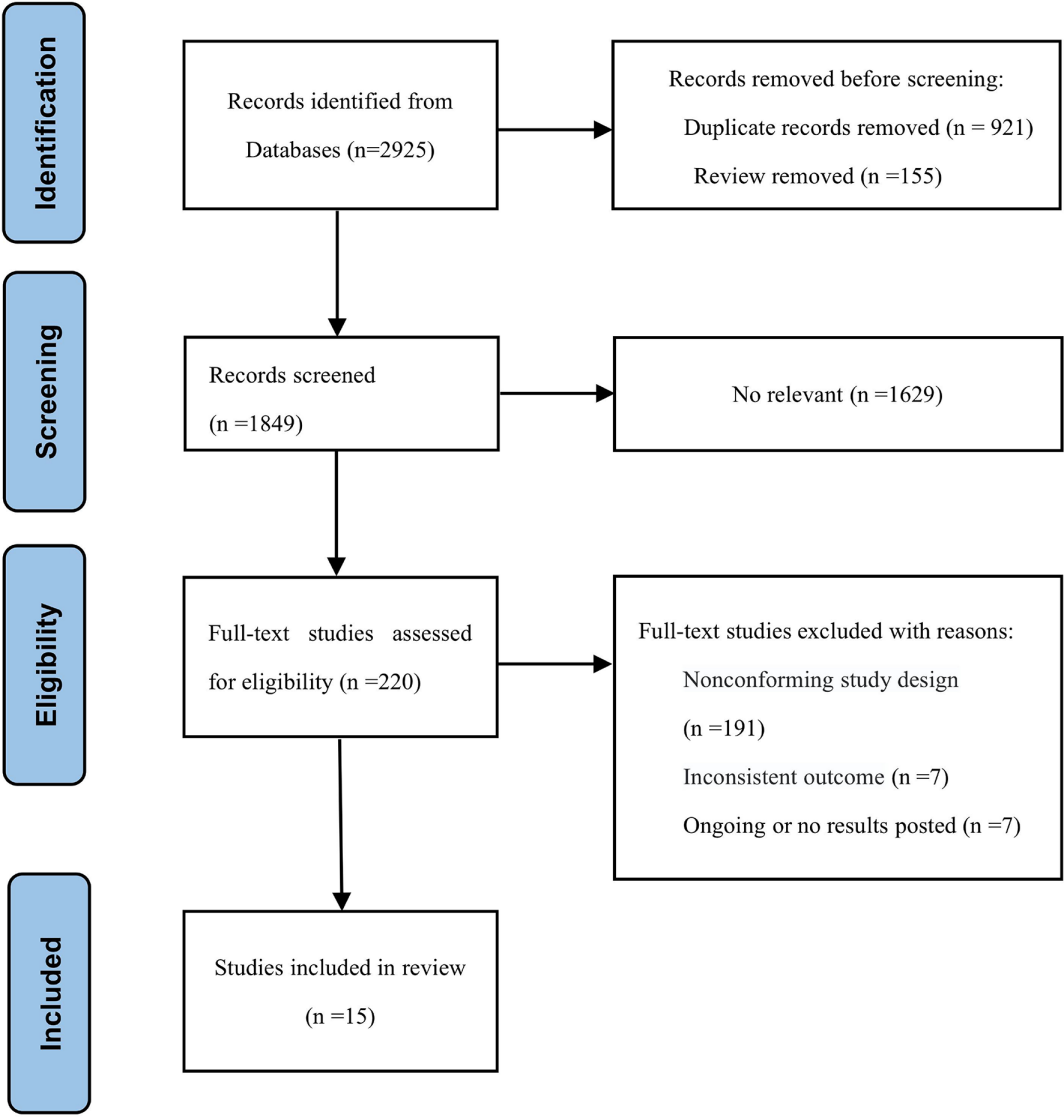
Flow chart of the studies selection process.

The search strategy identified 2925 citations. We reviewed the titles and abstracts and retrieved the full text of potential eligible research for inclusion. Finally, our systematic review and meta-analysis included 15 studies (6 RCTs and 9 retrospective studies). A flow chart of the literature screening is shown in **Figure 1**. A total of 6853 patients were included in our analysis, of which, 2948 were in the experimental group and 3905 were in the control group. The detailed characteristics of each research were summed up in **Table 1**.

**Table 1:** Characteristics of studies enrolled.

Study	Country	Study design	N (ADT + RT/RT)	Age/years		ADT+RT	RT	NOS score
				ADT+RT	RT			
Nabid, A 2021 (19)	Canada	RCT	200/200	71(54-80)	71(50-79)	(Bicalutamide 50mg/d + Goserelin 10.8mg/3 months, 6 months) + 3D-CRT: 76Gy	3D-CRT: 76Gy	–
Krauss, D. J 2021 (20)	American	RCT	742/750	–	–	(LHRH Agonists/Antagonists + Antiandrogen, 6 months) + EBRT: 79.2 Gy or (EBRT: 45 Gy + LDR/HDR -BT)	EBRT: 79.2 Gy or (EBRT: 45 Gy + LDR/HDR-BT)	–
Thorpe, C. S 2021 (21)	American	RCT	55/55	–	–	LHRH Agonists, 6 months + moderately hypofractionated PBT: 70Gy/28f	moderately hypofractionated PBT: 70Gy/28f	–
Vargas, C. E 2019 (22)	American	RCT	56/60	65.5(51-76)	68.3(49-76)	LHRH Agonists, 6 months + IMRT: 81Gy/45f or (IMRT: 45Gy/25f + BT: ^103^Pd 100 Gy)	IMRT: 81Gy/45f or (IMRT: 45Gy/25f + BT: ^103^Pd 100 Gy)	–
Dubray, B. M 2016 (23)	France	RCT	179/191	–	–	(Flutamide + Triptorelin, 4months) + EBRT: 80Gy/40f	EBRT: 80Gy/40f	–
Jones, C. U 2011 (24)	American	RCT	524/544	–	–	(Flutamide 750 mg/d or Goserelin 3.6mg/m or Leuprolide 7.5mg/m, 4 months) + EBRT: 66.6Gy/37f	EBRT: 66.6Gy/37f	–
Post, C 2022 (25)	American	Retrospective	94/166	67.3	66.9	(Bicalutamide 50mg/d and LHRH Agonists, 6 months) + IMRT: 78Gy/39f or 70Gy/35f	IMRT: 78Gy/39f or 70Gy/35f	7
Pickles, T 2017 (26)	Canada	Retrospective	121/139	67(52-83)	68(43-84)	ADT, 6 months + LDR- BT	LDR-BT	6
Dong,Y 2017 (16)	American	Retrospective	155/979	–	–	ADT + 3D-CRT/IMRT: 74-80Gy/70.2Gy	3D-CRT/IMRT: 74-80Gy/70.2Gy	6
Boladeras, A 2016 (27)	Spain	Retrospective	163/183	–	–	(Leuprorelin/Triptorelin/Goserelin + Bicalutamide) + 3D-CRT:< 76Gy (before 2000.08) or ≥76 Gy (after 2000.08)	3D-CRT:< 76Gy (before 2000.08) or ≥76 Gy (after 2000.08)	6
Schiffmann, J 2014 (28)	Germany	Retrospective	84/97	–	–	ADT + (3D-CRT: 50.4Gy/28f + HDR-BT)	3D-CRT: 50.4Gy/28f + HDR-BT	7
Schreiber, D 2014 (29)	American	Retrospective	62/141	70(49-85)		ADT + (3D-CRT (2003-2006)/IMRT (2006-2010)/IMRT + IGRT (after 2010), ≥75.6Gy)	3D-CRT (2003-2006)/IMRT (2006-2010)/IMRT + IGRT (after 2010), ≥75.6Gy	6
Cabeza Rodriguez, M 2013 (30)	Spain	Retrospective	40/145	–	–	ADT + 3D-CRT: 76Gy	3D-CRT: 76Gy	6
Edelman, S 2012 (31)	American	Retrospective	123/173	68.3	66.4	(LHRH Agonists, 4-6 months +/- Testosterone Receptor Antagonists, 1-4 months) + (IMRT≥72Gy +/- IGRT)	IMRT≥72Gy +/- IGRT	8
Stock, R. G 2010 (32)	American	Retrospective	350/82	–	–	(LHRH Agonists +/- Antiandrogen) + (3D-CRT/IMRT: 39.6-61.2Gy/22-34f + BT (^103^Pd 100Gy or ^125^I 125Gy))	3D-CRT/IMRT: 39.6-61.2Gy/22-34f + BT (^103^Pd 100Gy or ^125^I 125Gy)	8

ADT, androgen deprivation therapy; RT, radiotherapy; NOS, Newcastle–Ottawa scale; RCT, randomized controlled trial; 3D-CRT, 3-dimensional conformal radiation therapy; LHRH, luteinizing hormone-releasing hormone; EBRT, external beam radiotherapy; LDR, low-dose-rate; HDR, high-dose-rate; BT, brachytherapy; PBT, proton beam therapy; IMRT, intensity modulated radiation therapy; IGRT, image-guided radiation therapy.

### 3.2 Quality assessment

**Figure 2:**
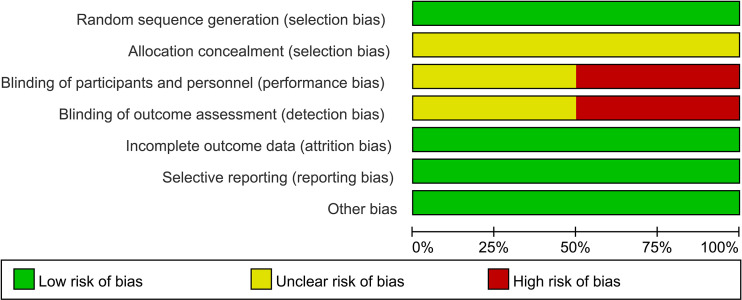
Risk of bias assessment for 6 RCTs.

**Figure 3:**
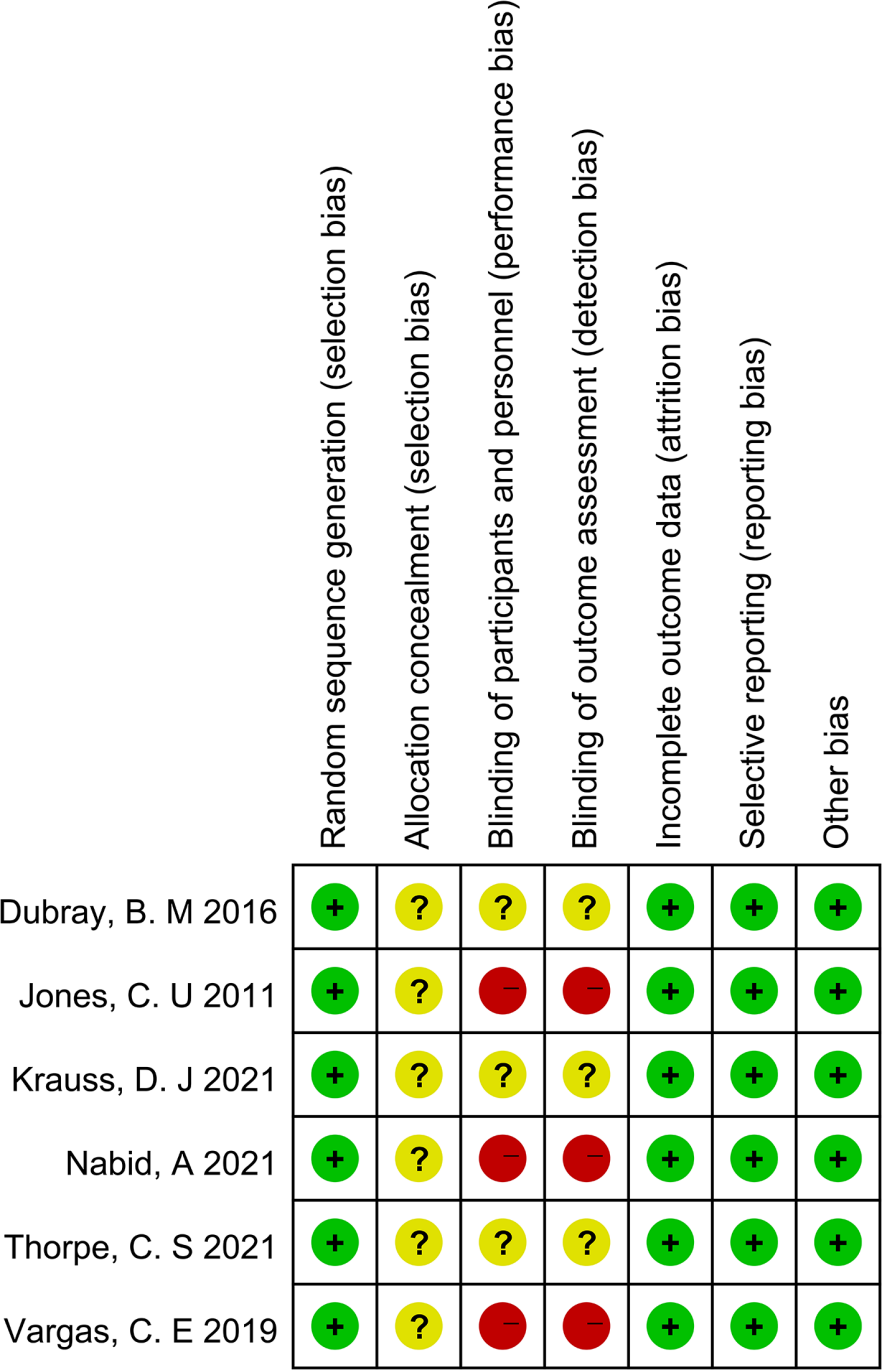
Risk of bias summary for 6 RCTs.

As shown in **Figures 2** and **3**, we evaluated the quality of the six final included RCTs using the Cochrane Collaboration’s tool. One RCT used the permuted-block randomization Method for random assignment, one used the hierarchical random assignment method, and the remaining four did not describe any particular randomization methods. None of the RCTs included provided sufficient information to evaluate the adequacy of allocation concealment. Three RCTs obtained informed consent from all included patients and were considered unblinded. The remaining three RCTs did not mention information about blindness. The NOS was also used to assess the included retrospective studies. The data and results reported in all included RCTs included were complete and without selective reporting or other bias. At the same time, the NOS was used to evaluate the retrospective studies, and the scores were all greater than or equal to 6. The evaluation results are shown in **Table 1**. Overall, all the studies included in this meta-analysis were considered to be of high quality.

### 3.3 Efficiency

#### 3.3.1 OS

There were 4 RCTs and 6 retrospective studies of the included articles reporting OS.

**Figure 4:**
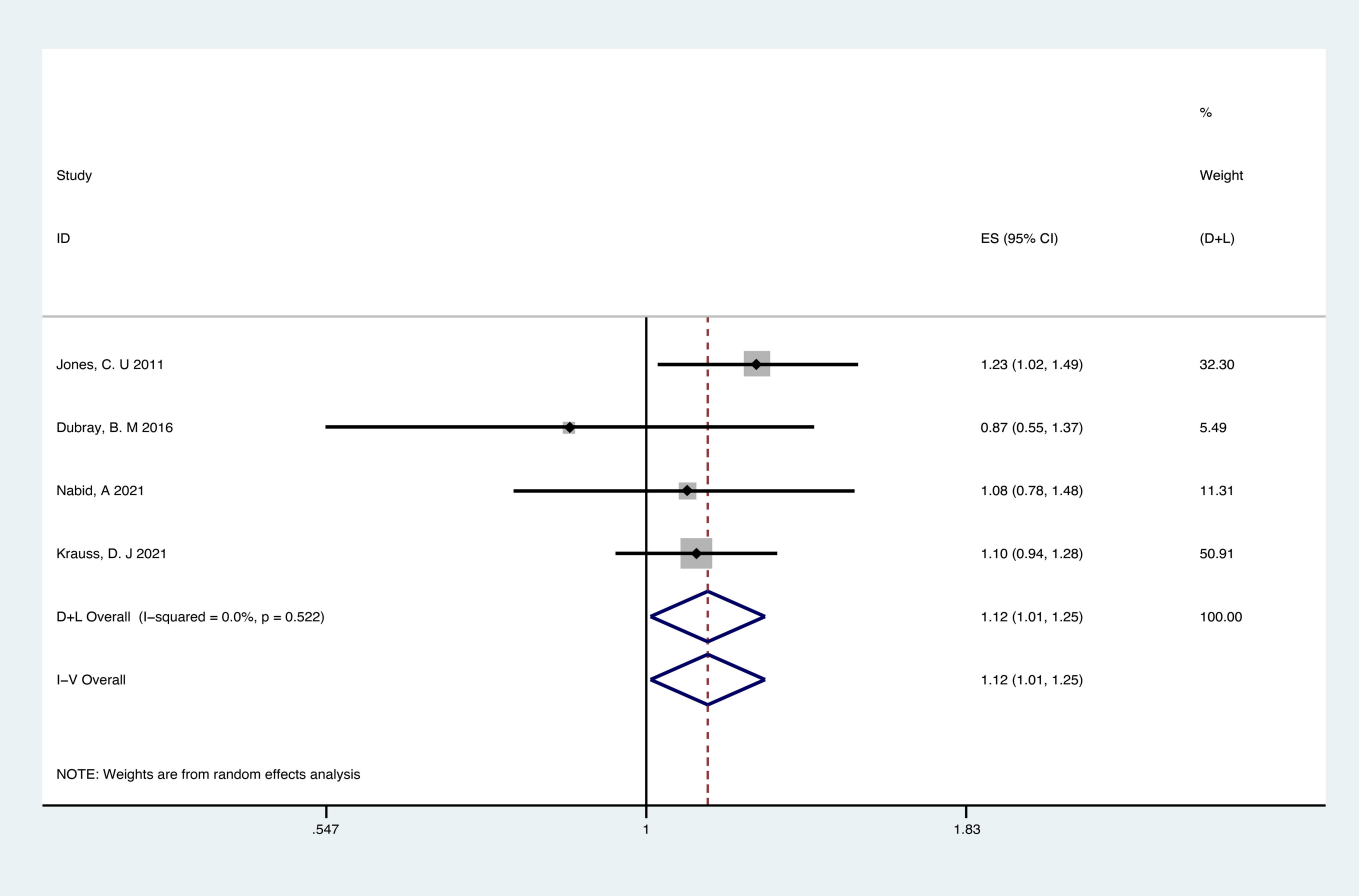
Forest plot for OS of 4 RCTs.

Analyses of RCTs for OS showed that RT plus ADT was associated with a 12% increase in the risk for all-cause mortality (ACM) (HR 1.12, 95% CI 1.01-1.12, p=0.04) with no heterogeneity between studies (P =0.522, I^2^ = 0%) and the results were statistically significant (**Figure 4**).

**Figure 5:**
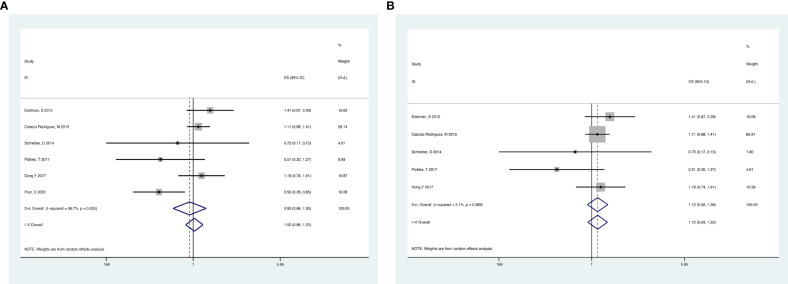
Forest plot for OS of 6 retrospective studies before the sensitivity analysis **(A)**, after the sensitivity analysis **(B)**.

**Figure 6:**
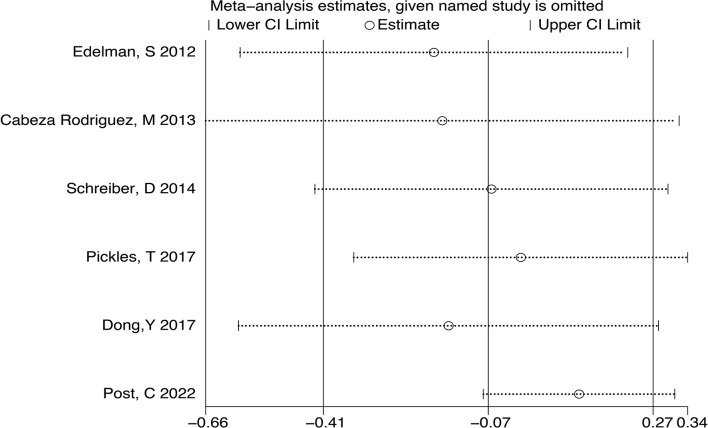
Sensitivity analysis for OS of 6 retrospective studies.

Analyses of the retrospective studies for OS showed that RT plus ADT was associated with a 2% increase in the risk for ACM (HR 1.02, 95% CI 0.86-1.22, P=0.79) with significant heterogeneity between studies (P =0.033, I^2^ =58.7%) (**Figure 5A**). Because of this, we tried to conduct the sensitivity analysis, which was performed by excluding one study at a time to assess the influence of each study on overall results. The results showed that the deletion of Post,C’s study had a significant effect on the results (**Figure 6**), so we exclude Post,C’s study and reanalyzed the remaining studies, which showed that RT plus ADT was associated with a 12% increase in ACM (HR 1.12, 95% CI 0.93-1.35, P=0.24) with no heterogeneity between studies (P =0.389, I^2^ =3.1%) (**Figure 5B**) but the results were no statistically significant.

#### 3.3.2 BCRFS

**Figure 7:**
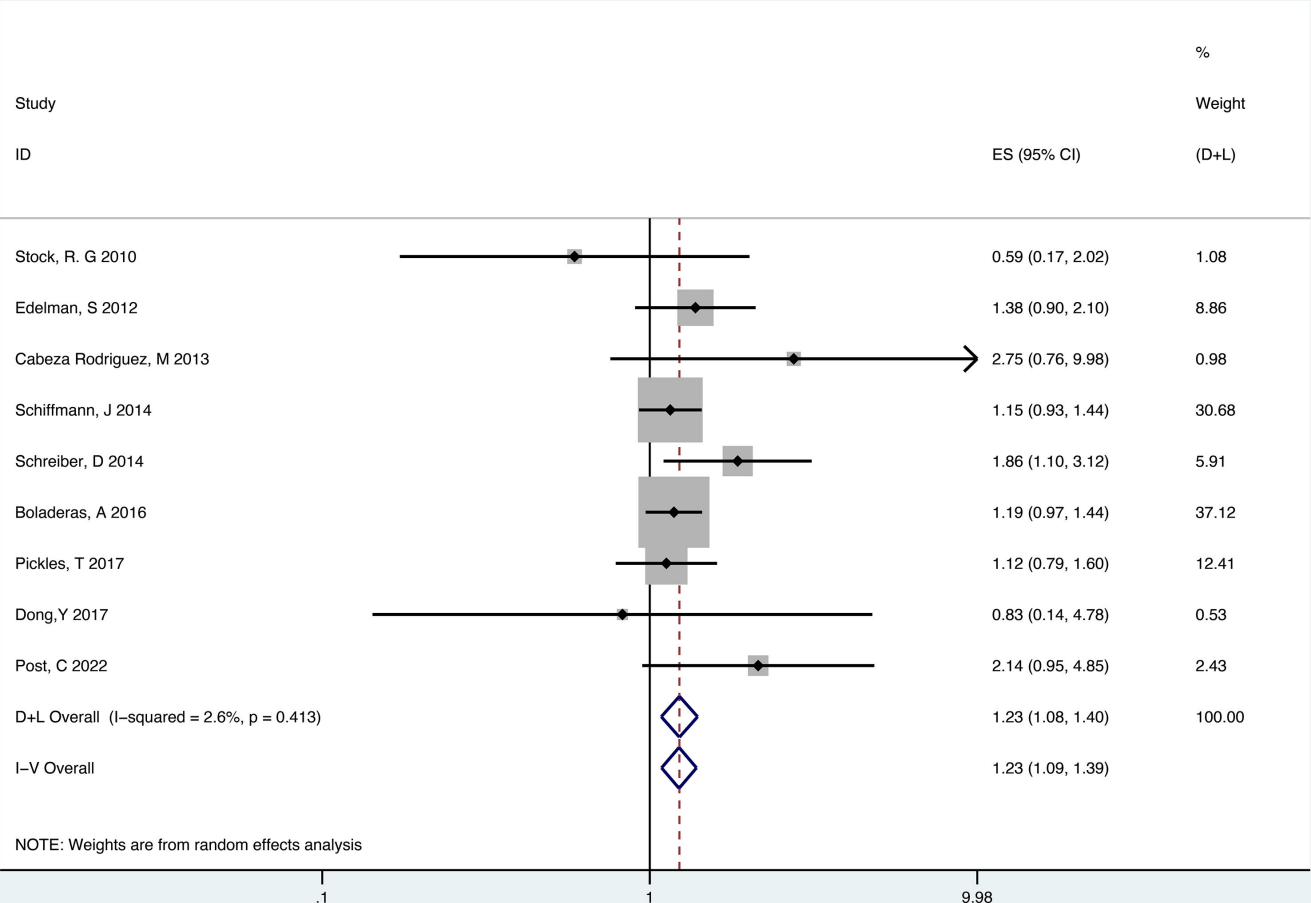
Forest plot for BCRFS of 9 retrospective studies.

There were 9 retrospective studies of the included articles reporting BCRFS. The results showed that RT plus ADT was associated with a 23% increase in the risk for ACM (HR 1.23, 95% CI 1.09-1.39, P=0.001) with no heterogeneity between studies (P =0.413, I^2^ = 2.6%) and the results were statistically significant (**Figure 7**).

### 3.4 Sensitivity and publication Bias evaluation

**Figure 8:**
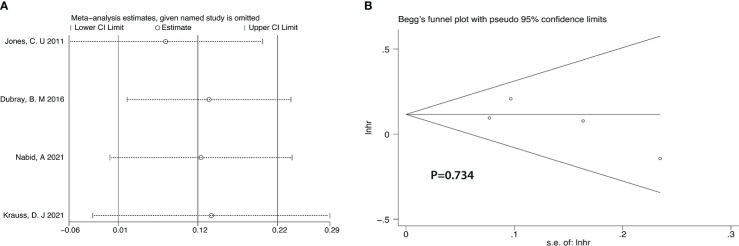
Sensitivity analysis **(A)** and Begg’s funnel plot **(B)** for OS of 4 RCTs.

**Figure 9:**
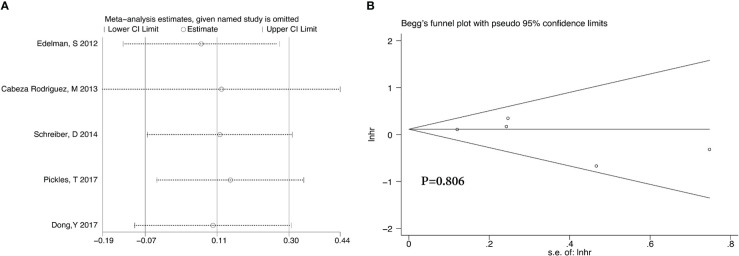
Sensitivity analysis **(A)** and Begg’s funnel plot **(B)** for OS of 5 retrospective studies.

**Figure 10:**
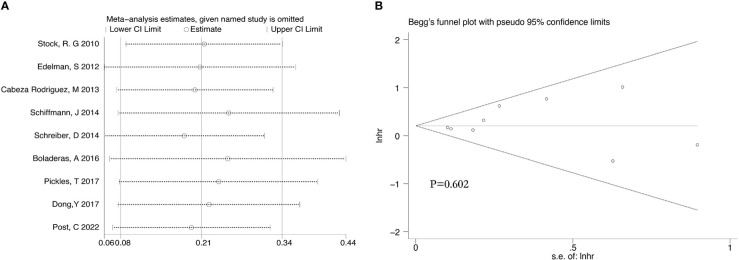
Sensitivity analysis **(A)** and Begg’s funnel plot **(B)** for BCRFS of 9 retrospective studies.

After we analyzed the OS and BCRFS of the included articles, we carried out the sensitivity analyses and the deletion of any one study had no significant effect on the results which showed our meta-analysis is relatively stable (**Figure 8A**-**10A**). We also conducted the publication bias evaluation and no evidence of publication bias was found based on Begg’s funnel plot (**Figure 8B**-**10B**).

## 4 Discussion

PC originates from prostatic intraepithelial neoplasia (PIN), which develops into high-grade PIN (HG-PIN), and eventually becomes adenocarcinoma (33, 34). At this point, the cancer cells have penetrated the epithelium and invaded the basal cells, and metastases will soon appear. The growth and function of prostate cells are highly dependent on androgen and inhibition of androgen production or androgen receptor(AR) can play an anticancer effect in all stages of PC (35). However, there are many complications including hyperlipidemia, abdominal obesity, diabetes mellitus type 2, cardiovascular disease, osteoporosis, and so on due to the decrease of androgen levels after ADT treatment (36–40). Therefore, it is important to recognize the hazards of ADT and select the most suitable patients to achieve the purpose of precision treatment.

PC is a malignant men’s tumor with high morbidity and mortality and has become an important public health issue (41). As mentioned above, ADT is the basic treatment for PC but the need for ADT combined with RT is controversial, especially in intermediate PC patients (42, 43). Early two prospective clinical research involving primarily intermediate PC patients showed the benefit of OS when added to 4 to 6 months of ADT before RT (12, 24). On the contrary, a randomized phase 3 study(PMH 9907) showed that the addition of ADT to RT did not significantly affect BF and OS (44). The reason for this difference may be the included patients were not all intermediate PC. As a result, it is needed for the clinical study aimed at intermediate PC patients accurately. Our meta-analysis included 15 studies, which were all based on the patients categorized as the intermediate risk and these targeted choices could make our research more significant.

The original intention of our study was to further divide intermediate-risk PC into FIR and UIR. However, after analyzing the included studies, we found that few studies conducted this subgroup analysis. Nabid A et al. carried out a randomized phase III trial (19), and there was no significant difference in OS between patients receiving DERT 76Gy alone, ADT plus RT 70Gy, and ADT plus DERT 76Gy. Then, they conducted the secondary analysis of the trial and found that UIR patients had significantly worse distant metastases-free survival (DMFS) and OS from ADT but for FIR patients, OS was not significantly different between arms (45). Zachary S et al. also conducted a secondary analysis of the RTOG 9408 randomized clinical trial,which showed that in patients with UIR, ADT improved DM and PCMS but in patients with FIR, ADT did not improve DM, PCMS, and ACM (46). The above two studies indicated that therapeutic optimization may appear possible in intermediate-risk PC patients, FIR patients seem to benefit from RT alone and UIR patients seem to ADT plus RT. Because of most of studies didn’t do these subgroup analyses from intermediate risk patient and intermediate-risk PC was a heterogeneous group which had variable prognoses, our study should have some certain reference significance for the treatment of this grey zone in clinical works.

This systematic review included 6 RCTs and 9 retrospective studies and we analyzed the data separately to improve the accuracy of the analysis result. The results of our study showed that ADT plus RT treatment did not improve the OS and BCRFS, on the contrary, it increased the risk for ACM for the patients of intermediate-risk PC. The most intriguing finding of our meta-analysis was that it was nearly reported in the early study that ADT plus RT treatment showed a clinical benefit in terms of OS and BCRFS. However, some recent studies did not show ADT plus RT treatment’s benefit in OS and BCRFS. It may have something to do with the lack of intensity-modulated radiotherapy (IMRT) and relative low doses therapy. With the improvement of radiotherapy technology and the increase in radiation dose, the absolute advantage of ADT decreased. RT alone, compared to ADT plus RT, maybe the more suitable therapy for intermediate-risk PC patients. However, further personalized therapy should be performed through more detailed studies.

## 5 Limitations

There are still certain limitations of our meta-analysis. First, there were inconsistencies in the patients’ RT and ADT regimens and it may cause some degree of bias. Second, although we separately analyzed the RCTs and retrospective studies and got the same result that ADT plus RT was not improving OS and BCRFS compared with RT alone, the number of RCTs we enrolled was still small (only 4 studies). Third, the increased risk of ACM in patients receiving ADT may be directly attributed to the use of hormonal therapy but also other co-factors such as comorbidities or incorrect patient selection, which may be the bias. Finally, we used different approaches to extract the data we needed, especially the data of OS and which may bring some bias.

## 6 Conclusions

Our present meta-analysis revealed that ADT plus RT did not provide OS or BCRFS advantage to intermediate-risk PC patients. RT alone was an adequate therapeutic regimen for intermediate-risk PC patients. However, more detailed therapy methods, whether the favorable risk group or the unfavorable risk group patients should be treated with the same therapy regimen remain need some large clinical trials to confirm.

## Data availability statement

The original contributions presented in the study are included in the article/supplementary material. Further inquiries can be directed to the corresponding authors.

## Author contributions

JC, FZ and YX were responsible for comprehensive study design, paper revision, and submission. YY, MF and YZ contributed to the search of articles and data extraction. JC, XS and YL performed the statistical analysis. All authors contributed and approved the submitted version.
