# Supplementary material for: Androgen deprivation therapy and radiotherapy in intermediate-risk prostate cancer: A systematic review and meta-analysis
Source: Front Endocrinol (Lausanne). 2023 Jan 17;13:1074540. doi: 10.3389/fendo.2022.1074540 (PMC9887024; doi:10.3389/fendo.2022.1074540)
Supplement: Supplementary file 1 [file DataSheet_1.docx]

Supplementary Material

# Supplementary materials caption

**Figure S1** | Funnel plot of the analysis for OS of 4 RCT(A) and 5 retrospective studies after the sensitivity analysis(B).

**Figure S2** | Egger’s plot of the analysis for OS of 4 RCTs(A) and 5 retrospective studies after the sensitivity analysis(B).

**Figure S3** | Egger’s tests for publication bias for BCRFS of 9 retrospective studies.

**Figure S4** | Begg’s and Egger’s tests for publication bias for OS of 4 RCT(A) and 5 retrospective studies after the sensitivity analysis(B) and for BCRFS of 9 retrospective studies(C).


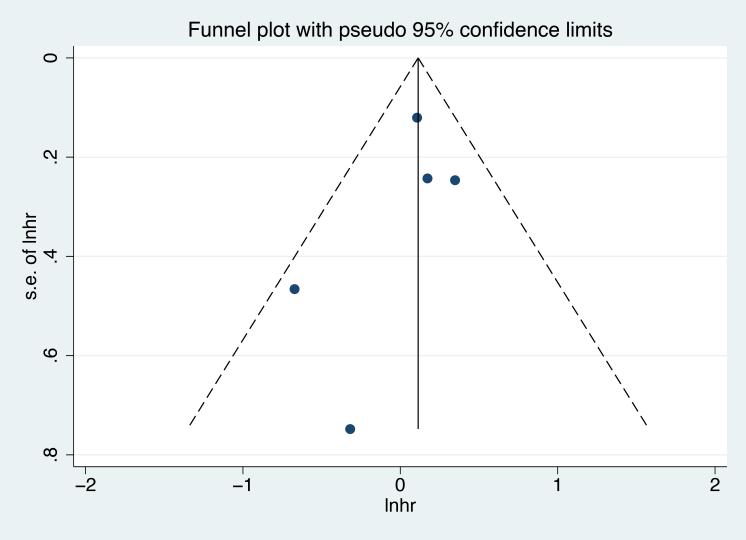

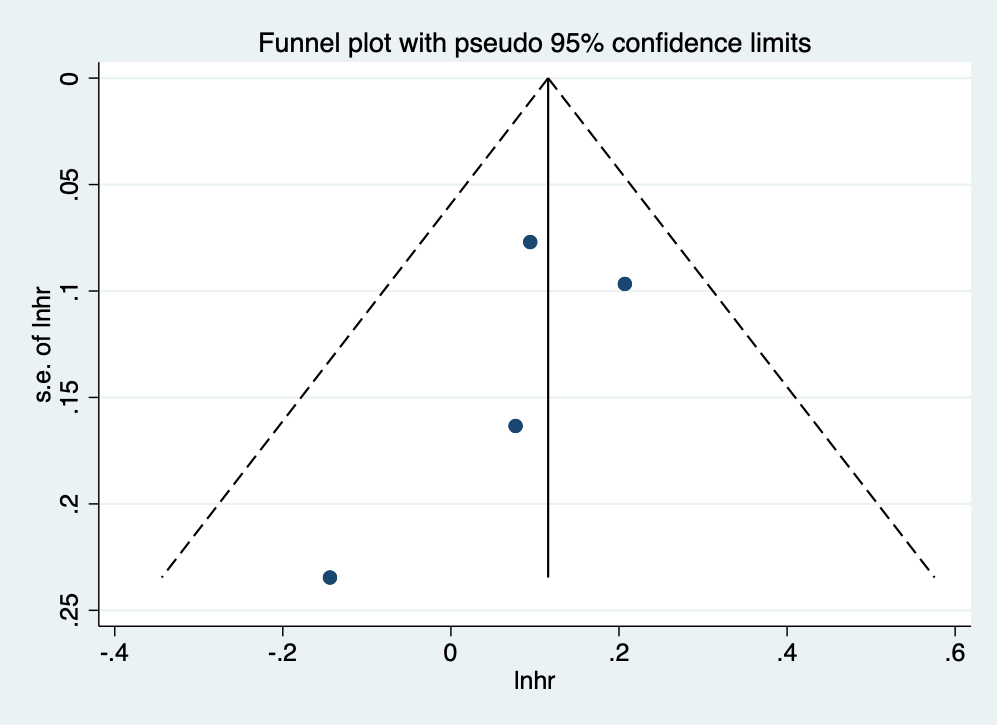
A B

Figure S1 | Funnel plot of the analysis for OS of 4 RCT(A) and 6 retrospective studies after the sensitivity analysis(B).

A B


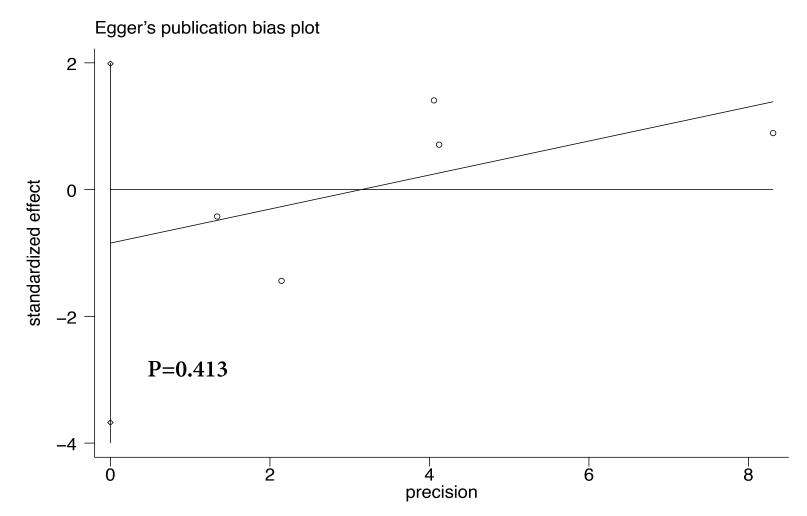

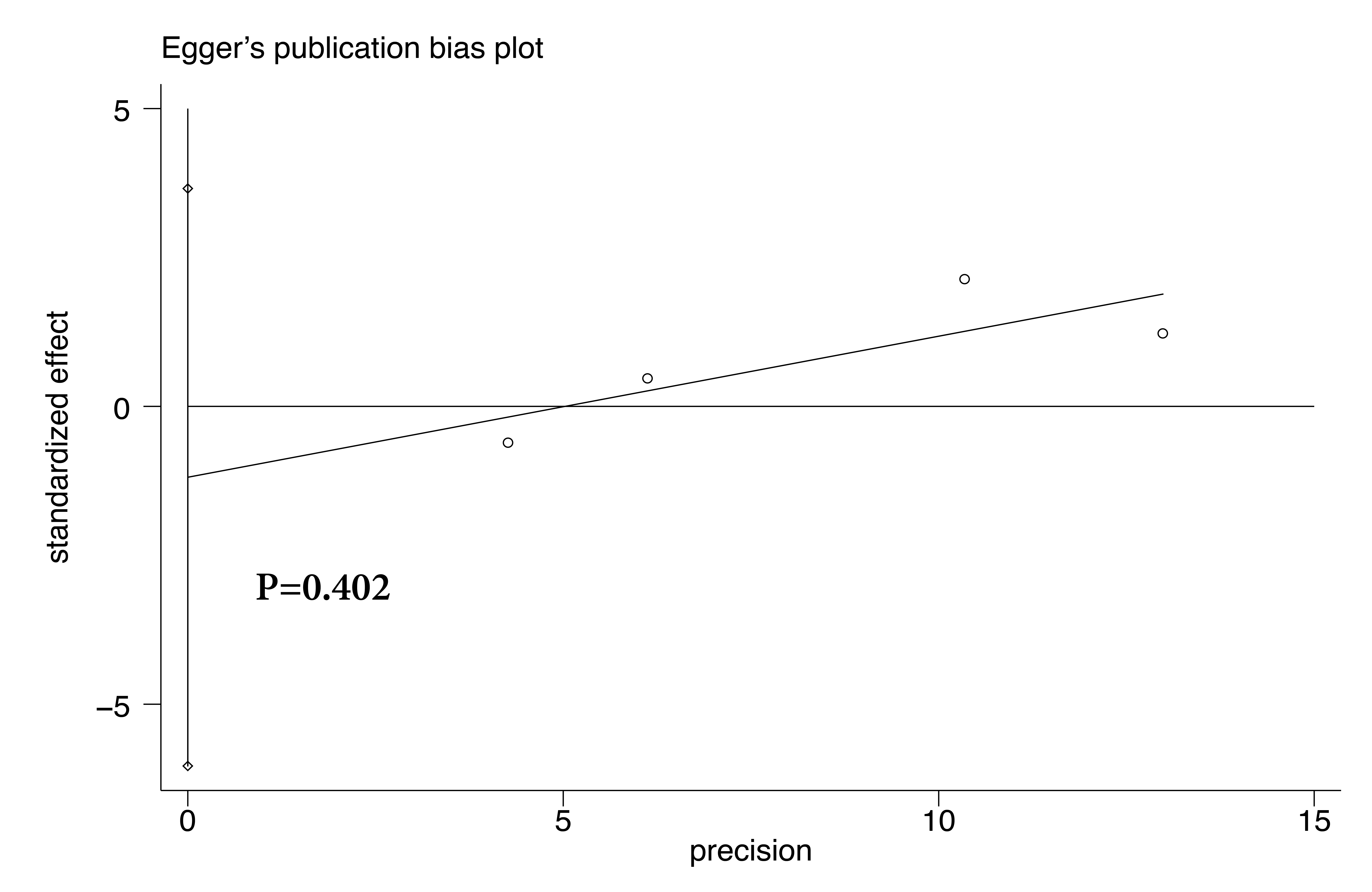


**Figure S2** | Egger’s plot of the analysis for OS of 4 RCTs(A) and 5 retrospective studies after the sensitivity analysis(B).


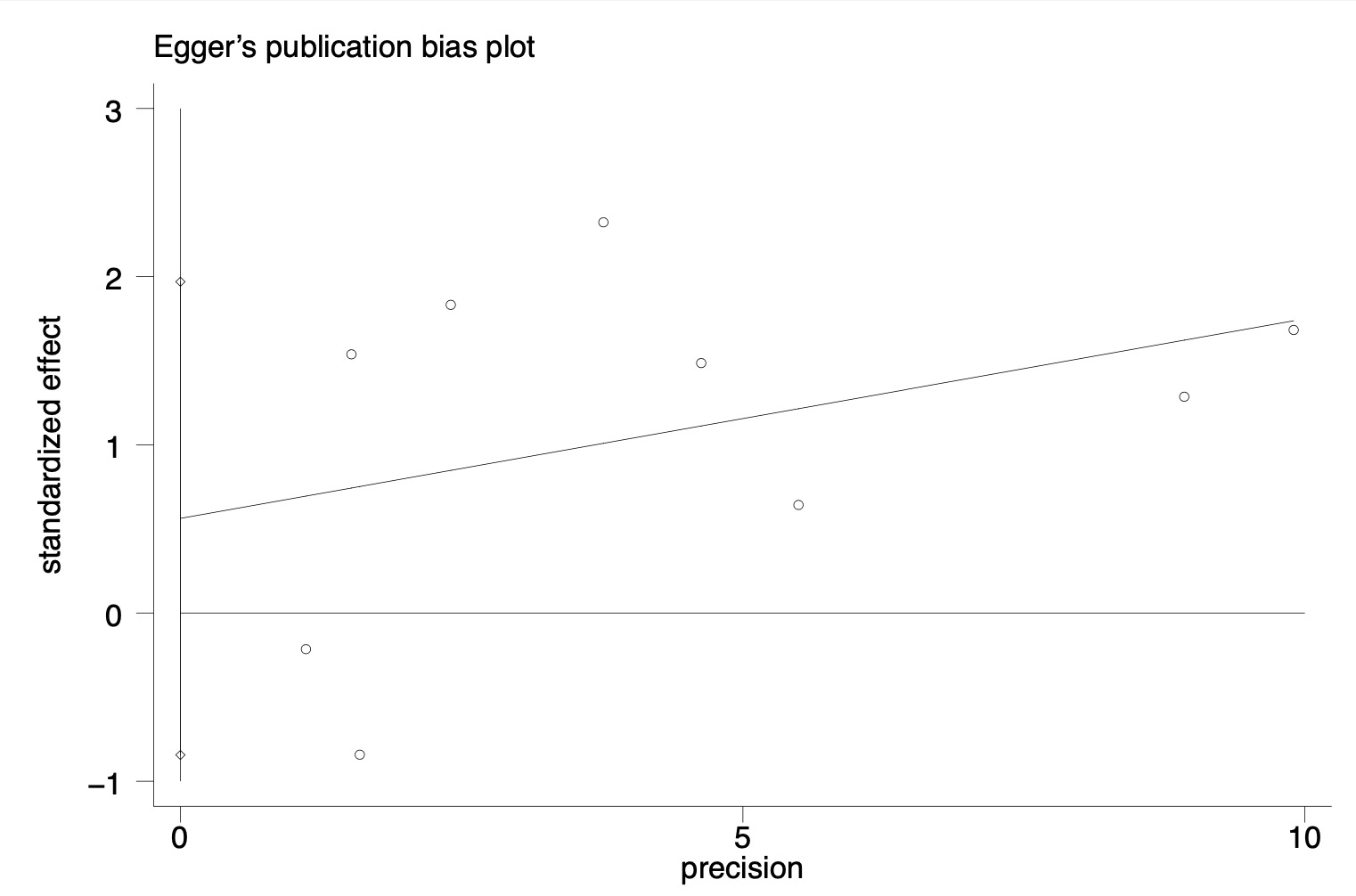


**Figure S3** | Egger’s tests for publication bias for BCRFS of 9 retrospective studies.


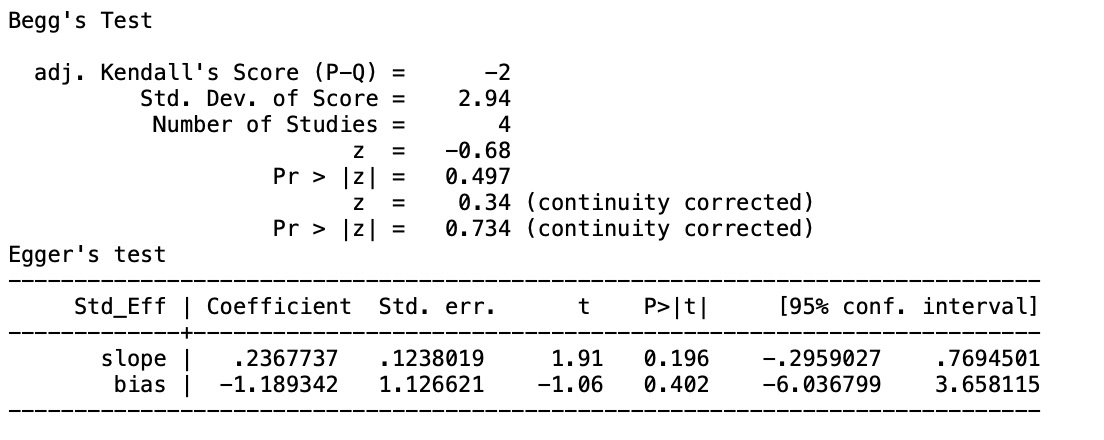
**A**

**B**


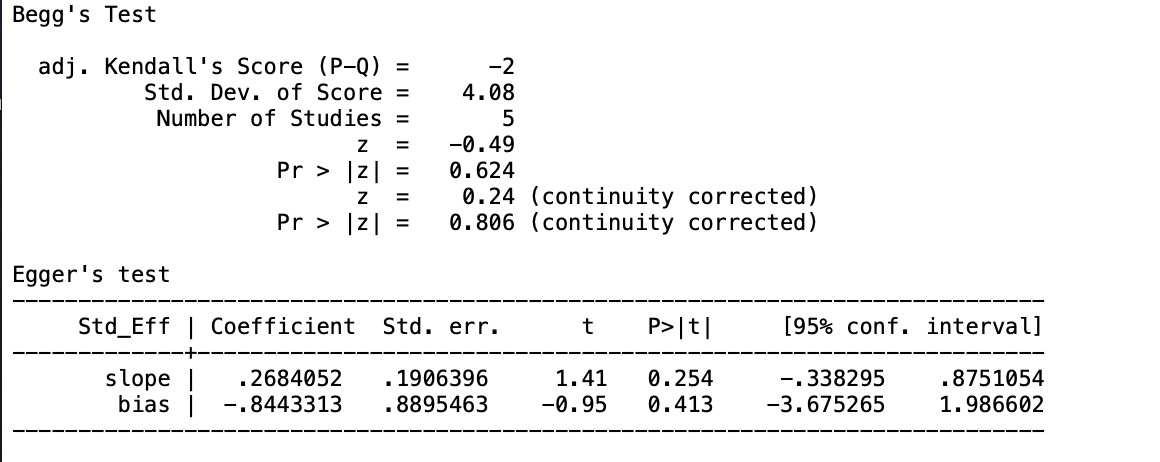


**C**


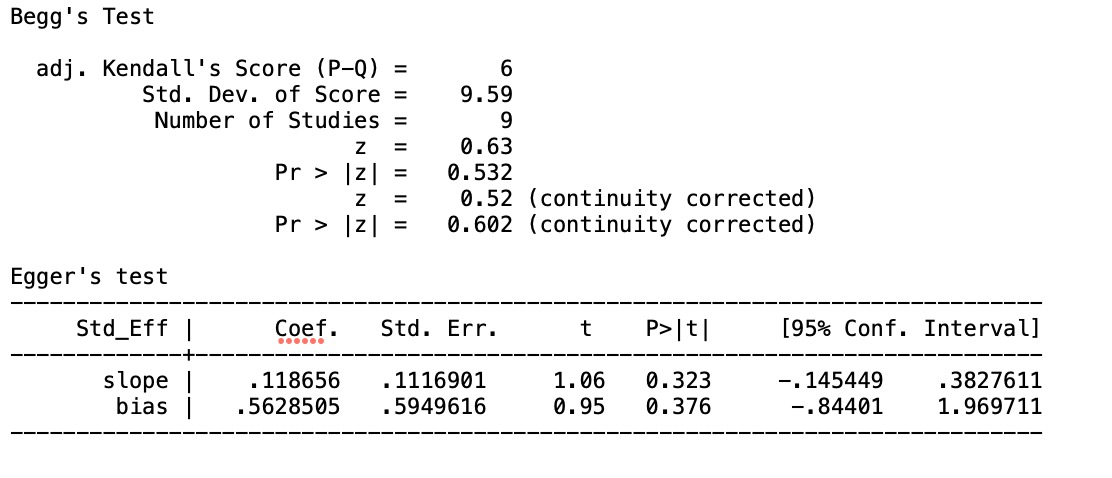


**Figure S4** | Begg’s and Egger’s tests for publication bias for OS of 4 RCT(A) and 5 retrospective studies after the sensitivity analysis(B) and for BCRFS of 9 retrospective studies(C).

**
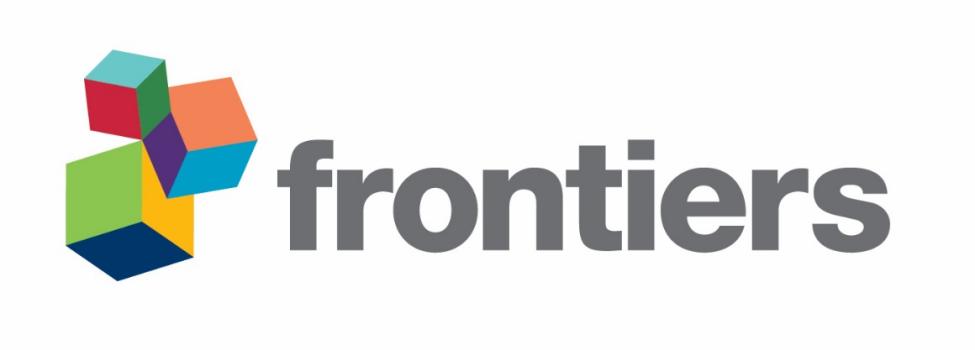
**
